# *In situ*/*operando* plug-flow fixed-bed cell for synchrotron PXRD and XAFS investigations at high temperature, pressure, controlled gas atmosphere and ultra-fast heating

**DOI:** 10.1107/S1600577523009591

**Published:** 2024-01-01

**Authors:** Benjamin Bischoff, Maged F. Bekheet, Emiliano Dal Molin, Sebastian Praetz, Birgit Kanngießer, Reinhard Schomäcker, Martin Etter, Henrik S. Jeppesen, Akhil Tayal, Aleksander Gurlo, Albert Gili

**Affiliations:** a Technische Universität Berlin, Faculty III Process Sciences, Institute of Materials Science and Technology, Chair of Advanced Ceramic Materials, Straße des 17 Juni 135, 10623 Berlin, Germany; b Technische Universität Berlin, Faculty III Process Sciences, Institute for Optic and Atomic Physics, Straße des 17 Juni 135, 10623 Berlin, Germany; c Technische Universität Berlin, Faculty II Mathematik und Naturwissenschaften, Institut für Chemie, Straße des 17 Juni 135, 10623 Berlin, Germany; d Deutsches Elektronen-Synchrotron (DESY), Notkestrasse 85, 22607 Hamburg, Germany; e Helmholtz-Zentrum Berlin für Materialien und Energie, 14109 Berlin, Germany; University of Malaga, Spain

**Keywords:** plug-flow fixed-bed cell, ultra-fast infra-red heating, gas–solid interactions, *in situ* X-ray diffraction, *in situ* X-ray absorption fine structure

## Abstract

A plug-flow fixed-bed cell for synchrotron powder X-ray diffraction (PXRD) and X-ray absorption fine structure (XAFS) idoneous for the study of heterogeneous catalysts at high temperature, pressure and under gas flow is designed, constructed and demonstrated. The operating conditions up to 1000°C and 50 bar are ensured by a set of mass flow controllers, pressure regulators and two infra-red lamps that constitute a robust and ultra-fast heating and cooling method. The performance of the system and cell for carbon dioxide hydrogenation reactions under specified temperatures, gas flows and pressures is demonstrated both for PXRD and XAFS at the P02.1 (PXRD) and the P64 (XAFS) beamlines of the Deutsches Elektronen-Synchrotron (DESY).

## Abstract 2

A plug-flow fixed-bed cell for synchrotron powder X-ray diffraction (PXRD) and X-ray absorption fine-structure (XAFS) idoneous for the study of heterogeneous catalysts at high temperature, pressure and under gas flow is designed, constructed and demonstrated. The operating conditions up to 1000°C and 50 bar are ensured by a set of mass flow controllers, pressure regulators and two infra-red lamps that constitute a robust and ultra-fast heating and cooling method.

## 1. Introduction

The study of heterogeneous catalysts using synchrotron-based *in situ* techniques is at the core of rational catalyst design. A detailed picture of a catalytic system can be provided by a combination of electron-based (mainly electron microscopy) and photon-based methods like X-ray diffraction (XRD), X-ray photoelectron spectroscopy (XPS) and X-ray absorption fine structure (XAFS) (Bergmann & Roldan Cuenya, 2019 ▸). Comparing physical and chemical data with the performance in terms of reaction rates, selectivity and stability allows for elucidating reaction mechanisms, the active site and the deactivation mechanisms (Gili *et al.*, 2018 ▸; Bonmassar *et al.*, 2020 ▸). Establishing the structure–activity correlations is impossible without accessing structural information of the working catalysts under real operating conditions.

In order to perform *in situ* investigations at a beamline, one must ensure proper interaction of the target materials with the reactive atmosphere at a controlled and homogeneous temperature and pressure while the materials are exposed to a beam of photons. Thus, the design and operation of such tools is not trivial. The choice of materials is of uttermost importance: they must be capable of holding the desired reaction conditions while being inert and allowing the transmission of photons.

Several cells for powder X-ray diffraction (PXRD) or XAFS have been developed at different synchrotrons and allow for a wide variety of testing conditions (Andrieux *et al.*, 2014 ▸; Hansen *et al.*, 2015 ▸; Jensen *et al.*, 2010 ▸; Schlicker *et al.*, 2018*a* ▸; Pandit *et al.*, 2022 ▸). Static gas and dead-end configurations (Schlicker *et al.*, 2018*a* ▸) do not grant realistic gas–solid interactions as usually occur on plug-flow fixed-bed cells (Clausen *et al.*, 1991 ▸). Moreover, it is highly desirable to achieve elevated pressures, especially for CO_2_ hydrogenation reactions.

The current work describes the design, construction and demonstration of a plug-flow fixed-bed setup and cell for the study of heterogeneous catalysts at elevated temperature, pressure and gas atmosphere. This cell is a continuation of our work done previously at the Advanced Light Source of the LBNL, Berkeley, USA (Schlicker *et al.*, 2018*a* ▸; Doran *et al.*, 2017 ▸). It is upgraded in terms of the flow model (plug-flow) and the reachable pressure. The heating system, performed with a SiC tube acting as a furnace and two infra-red (IR) lamps, provides an extremely fast and robust heating method (Doran *et al.*, 2017 ▸). The cell is demonstrated regarding (i) temperature using Pt and Al_2_O_3_ calibrants, (ii) pressure using a Mg(OH)_2_ de­hydroxy­lation and carbonation reaction and (iii) for both *in situ* XAFS and PXRD following structural changes of a PdO/rh-In_2_O_3_ system under hydrogen flow (where rh indicates the rhombohedral form). The main goal of the current cell is to provide *in situ* structural data of heterogeneous catalysts in general: we have selected CO_2_ hydrogenation reactions as case examples due to their importance for the energy transition. The cell has been operated at two different beamlines of the PETRA III synchrotron at DESY, Hamburg, Germany: the P02.1 (PXRD) (Dippel *et al.*, 2015 ▸) and the P64 (XAFS) (Caliebe *et al.*, 2019 ▸) beamlines.

## 2. Equipment design and implementation

### 2.1. General technical description

**Figure 1:**
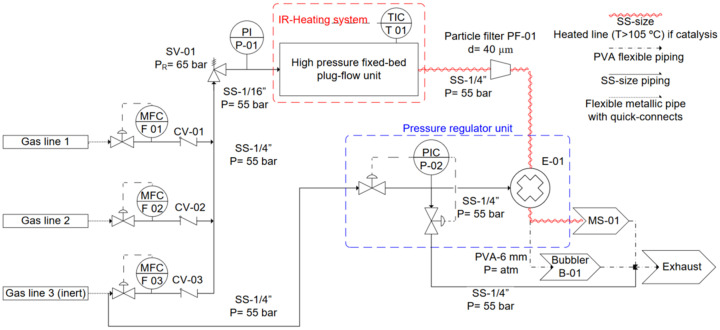
P&ID of the setup. All pressures are in bar absolute. MFC: mass flow controller; CV: check valve; SV: safety valve; PI: pressure indicator; SS: stainless steel; TIC: temperature indicator and controller; PIC: pressure indicator and controller; E: equilibar; MS: mass spectrometer.

The overall system is composed of (i) the upstream (gas flow control system), (ii) the reaction cell and (iii) the downstream. The pipes and instrumentation diagram (P&ID) can be seen in Fig. 1 ▸.

The upstream (i) contains three EL-FLOW Prestige mass flow controllers (MFCs 01–03; Bronkhorst, The Netherlands) connected to gas cylinders that enable the precise control of reactant gas composition and flow rate. Check-valves (CVs 01–03; Swagelok, USA) downstream of the MFCs prevent backward diffusion of gas. A digital pressure indicator (P-01; Omega, UK) allows monitoring of the pressure during the experiments. A stainless-steel high-pressure proportional relief valve (SV-01; Swagelok, USA) set to a burst pressure of 65 bar upstream of the plug-flow unit enables safe ventilation of the gas mixture to the exhaust should the system exceed its maximum pressure (*e.g.* through a sudden increase in reaction pressure due to chemical reaction). The reaction cell (ii) is described below in detail. The downstream (iii) of the system is composed of a particle filter with a grit of 40 µm (PF-01; FTSS series tee-type, Fitok, Germany), which prevents particles from moving downstream, potentially damaging the pressure regulator. Pressure control is enabled by a combination of an EL-PRESS pressure regulator (PIC P-02; Bronkhorst, The Netherlands) and an Equilibar research series back-pressure regulator (E-01; Equilibar, USA) limited to a pressure of 103.4 bar (1500 psi) and 150°C. The reason for using this combination for pressure regulation is to be able to heat up (∼105°C) the piping between the cell and the mass spectrometer (MS), thereby avoiding/reducing condensation of light compounds, such as methanol or water. It is important to note that P-02 does not measure or control (directly) the pressure inside the cell, it only measures and controls the pressure that E-01 receives, thus, the use of PI P-01 was necessary to read the real pressure in the cell and monitor potential leaks. An MS or another analytic device can be connected to obtain quantitative data on the performance of the catalysts. A video-surveyed bubble counter (B-01) provides visual confirmation of the gas flow. Both the analytics and bubbler outlets are directed toward the exhaust. All system parameters are controlled and recorded using a self-made Labview program (Bitter *et al.*, 2006 ▸).

### 2.2. Plug-flow fixed-bed reactor cell

**Figure 2:**
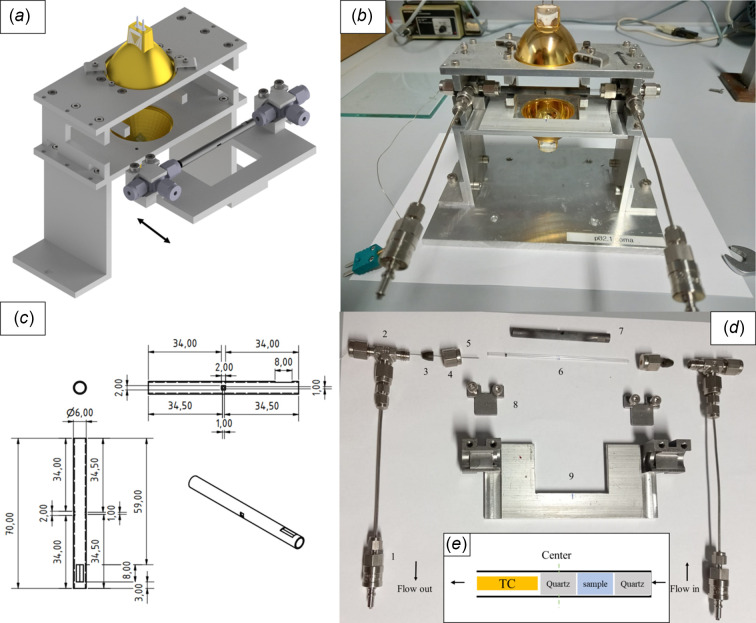
Schematic sketch and images of the cell: (*a*) CAD drawing of the cell, with the transport plate not assembled, (*b*) the assembled real cell, (*c*) details of the geometry and cuts in the SiC tube acting as furnace, (*d*) the disassembled parts of the cell, (*e*) schematic sketch of the sample, quartz wool and TC position inside the tube.

The cell design was similar to some previously applied for diffraction studies (Andrieux *et al.*, 2014 ▸). The sample is placed inside a fused silica tube [part 6 in Fig. 2 ▸(*d*), quartz glass; Hilgenberg, Germany] or a sapphire single-crystal tube (kindly provided by Crytur, Czech Republic). Both materials are reported to be extremely inert for the reactions of interest: amorphous quartz tubes are preferred as they do not add the typical diffraction spot of sapphire single crystals on the detector during PXRD. The tubes are opened at both ends, enabling a plug-flow mode inside the reactor cell. Dimensions of the tubing are 100 ± 0.5 mm in length, 1 ± 0.1 mm inner diameter and 1.5 ± 0.1 mm outer diameter (see §2.4 for an explanation of the sizing). The positioning of the sample inside the tube can be schematically seen in Fig. 2 ▸(*e*). At the exact center of the length of the tube, a 1 mm-long quartz wool segment is placed. Upstream of this quartz wool segment, 1–2 mm of the sample is placed, followed by a second 1 mm quartz wool segment. Downstream of this ‘quartz wool – sample – quartz wool’ assembly, a 0.5 mm-diameter thin K-type thermocouple (TC; Reckmann, Germany) is placed [5 in Fig. 2 ▸(*d*)]. The symmetry of this design places the TC tip and the sample at the exact same distance from the center of the tube, minimizing temperature differences. The hot TC can catalytically induce gas phase composition changes; to ensure controlled gas composition exposure of the sample, the TC is placed downstream of the sample. Finally, the TC also helps to hold the sample in position, although sample displacement can be avoided by mitigating sudden increases in gas flows. Mixed vespel/graphite ferrules [3 in Fig. 2 ▸(*d*); Mascom, Germany) are drilled to enclose the tube and seal it using Swagelok nuts [4 in Fig. 2 ▸(*d*)] and two T-pieces from Swagelok [2 in Fig. 2 ▸(*d*)]. These are plugged into Swagelok quick connects [1 in Fig. 2 ▸(*d*)], which allow the cell to be connected to the setup’s piping without screwing, preventing the tube breaking by torque. The total weight of the cell [with the support plate, Fig. 2 ▸(*b*), bottom big metal place with ‘p02.1 coma’ inscription] is 1.5 kg. Sample changes of the cell assembly are made by detaching the transport plate [Fig. 2 ▸(*a*), see arrow; 9 in Fig. 2 ▸(*d*)]. Two small metal plates [8 in Fig. 2 ▸(*d*)] fix the montage on the transport plate and prevent the tube from breaking during transport.

External to the reactor tube, a SiC tube acting as a furnace is assembled [Fig. 2 ▸(*c*), 7 in Fig. 2 ▸(*d*), original un­modified tubing kindly provided by Fraunhofer IKTS]. Note that this tube does not withstand any gas pressure from the cell. Two holes (X-ray entrance 1 mm × 1 mm and exit 2 mm × 2 mm) were cut with diamond cutting disks with sizes [see Fig. 2 ▸(*c*)] designed to allow for the X-ray beam to interact with the sample and travel toward the detector without interacting with the SiC tube. Two metallic U-shaped pieces [Fig. 2 ▸(*b*)] hold the SiC tubing, and rotation of the tube is prevented by softly placing the tip of a screw on top of a flat indent made on the SiC tubing of 8 mm in length [Fig. 2 ▸(*c*)]. SiC is the ideal material to act as a furnace due to its hardness and absorption coefficient (Schlicker *et al.*, 2018*a* ▸; Doran *et al.*, 2017 ▸). It heats up by being illuminated with two 64635 HLX halogen lamps (Osram, Germany). The wall of the SiC is placed at the focal point of the lamp (19.5 mm), and the TC is used for the control loop. A schematic sketch of the cell can be seen in Fig. 2 ▸(*a*), and in the explosion views in Figs. S1 and S2 of the supporting information.

### 2.3. Temperature measurements at the sample position

**Figure 3:**
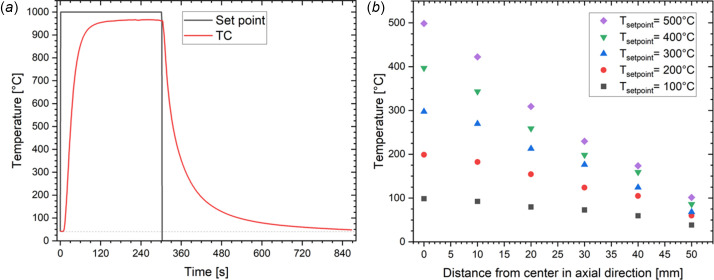
(*a*) The cell’s temperature response (TC) tracking the set point (rt to 1000°C and 1000°C to rt), performed under a pressure of 50 bar (abs) and a N_2_ flow rate of 5 Nml min^−1^. (*b*) The temperature profile from the center in the axial direction for different set points.

Fig. 3 ▸(*a*) shows the temperature measured by a TC placed at the sample position in the cell as a function of time at 50 bar (abs) and a constant N_2_ flow rate of 5 Nml min^−1^. A set point of 1000°C was selected, and the initial heating rate [for the room-temperature (rt) to 500°C range] shows the maximum heating rate of the setup, approximately 20°C s^−1^. As the temperature of the cell increases, so does the heat loss, and the heating rate progressively decreases until the cell reaches 965°C. Repeated use of the SiC tube progressively oxidizes the SiC, decreasing its absorption coefficient. Using a new SiC tube allows a temperature of 1000°C to be reached (see Fig. S3 of the supporting information). After a few seconds, the set point was selected to be 25°C and the temperature was recorded. Similarly, the maximum cooling rate is 20°C s^−1^ for the initial period. As no external cooling is applied, the cooling speed decreases as the temperature gradient between the cell and the environment decreases. This figure proves the ultra-fast heating and cooling concept of the cell. Fig. 3 ▸(*b*) shows the T-profile in the axial direction of the tube. It was achieved by displacing a second TC while maintaining the first TC (used for the set-point) in the center of the tube. We assume that these T-profiles are symmetric in the other axial direction.

### 2.4. Safety considerations

The fused silica tube sizing and wall thickness were calculated by applying the von Mises criterion using the same assumptions as Andrieux *et al.* (2014 ▸) and applying a safety factor of ×10. The robustness of several tubes was assessed at different temperatures, pressures, heating and cooling rates, and pressurizing rates before using them in the synchrotron experiments.

The system’s safety measures include the following. The MFCs are normally closed; in case of malfunction, gas flow into the system would stop. The pressure regulator concept is normally open: in case of malfunction, it would open and prevent a build-up of uncontrolled pressure. Check valves are used to prevent back-mixing of the gas flows. The piping sizing is very small to minimize the total volume of gas inside the system. The total volume between the MFCs and the equilibar is ∼25 ml. The stainless-steel high-pressure proportional relief valve (SV-01) prevents the system from reaching pressures above a safety threshold of 65 bar. All standard system components (except the quartz tubes) are certified for the maximum working pressure multiplied by a safety factor of (at least) 1.3. Prior to the operation, a burst test was performed at 1.3× maximum operating pressure, and a leak test was performed at mild pressures (∼5 bar) prior to each single experiment.

The current cell was designed to perform heterogeneous catalysis with a special focus on CO_2_ hydrogenation: the design system operating window is summarized in Table 1 ▸.

### 2.5. Beamline description and data analysis (*Dioptas*, Rietveld refinement)

This system and cell were installed and tested on two different beamlines of the PETRA III synchrotron. All data shown in the present work were obtained using the quartz capillaries. The P02.1 beamline for powder diffraction and total scattering (Dippel *et al.*, 2015 ▸) was operated at 60 keV (0.207 Å) with a sample–detector distance of 1200 mm in center ring configuration on a Varex 4343CT area detector. The beam size used was 0.7 mm × 0.7 mm. The cell [Fig. 2 ▸(*b*)] is assembled on the beamline’s *XYZ* Huber stage to allow for fine alignment with respect to the beam and the SiC tube/sample. Note that the current design does not allow for rotation in any directions. The 2D images from the detector were integrated using *Dioptas* (Prescher & Prakapenka, 2015 ▸) software and plotted using *Origin (Pro)*, Version 2022 (OriginLab Corporation, Northampton, MA, USA). Rietveld refinement was performed using *FULLPROF* software (Rodriguez-Carvajal, 1990 ▸). The instrument broadening parameters and the sample–detector distance were calibrated using an LaB_6_ NIST 660b standard. All pressures are absolute.

The XAFS measurements were performed at the P64 beamline, DESY (Caliebe *et al.*, 2019 ▸). An Si(111) monochromator was used for scanning the energy. The XAFS measurements were performed in the fluorescence and transmission modes simultaneously. The transmission and fluorescence signals were recorded using an ionization chamber and passivated implanted planar silicon (PIPS) detector. The data displayed in this work were obtained in fluorescence mode. Before normalization measurement artifacts were removed manually. The normalization of the XAFS spectra was performed with the flattening algorithm of *ATHENA* (part of the *Demeter* software package) (Ravel & Newville, 2005 ▸) using a first-degree polynomial for the pre-edge line and a second-degree polynomial for the post-edge line.

## 3. Experimental results

The applicability of the cell was demonstrated on three different levels: (i) temperature, (ii) pressure and (iii) for both *in situ* XAFS and PXRD.

### 3.1. Thermal expansion of platinum and alumina

**Figure 4:**
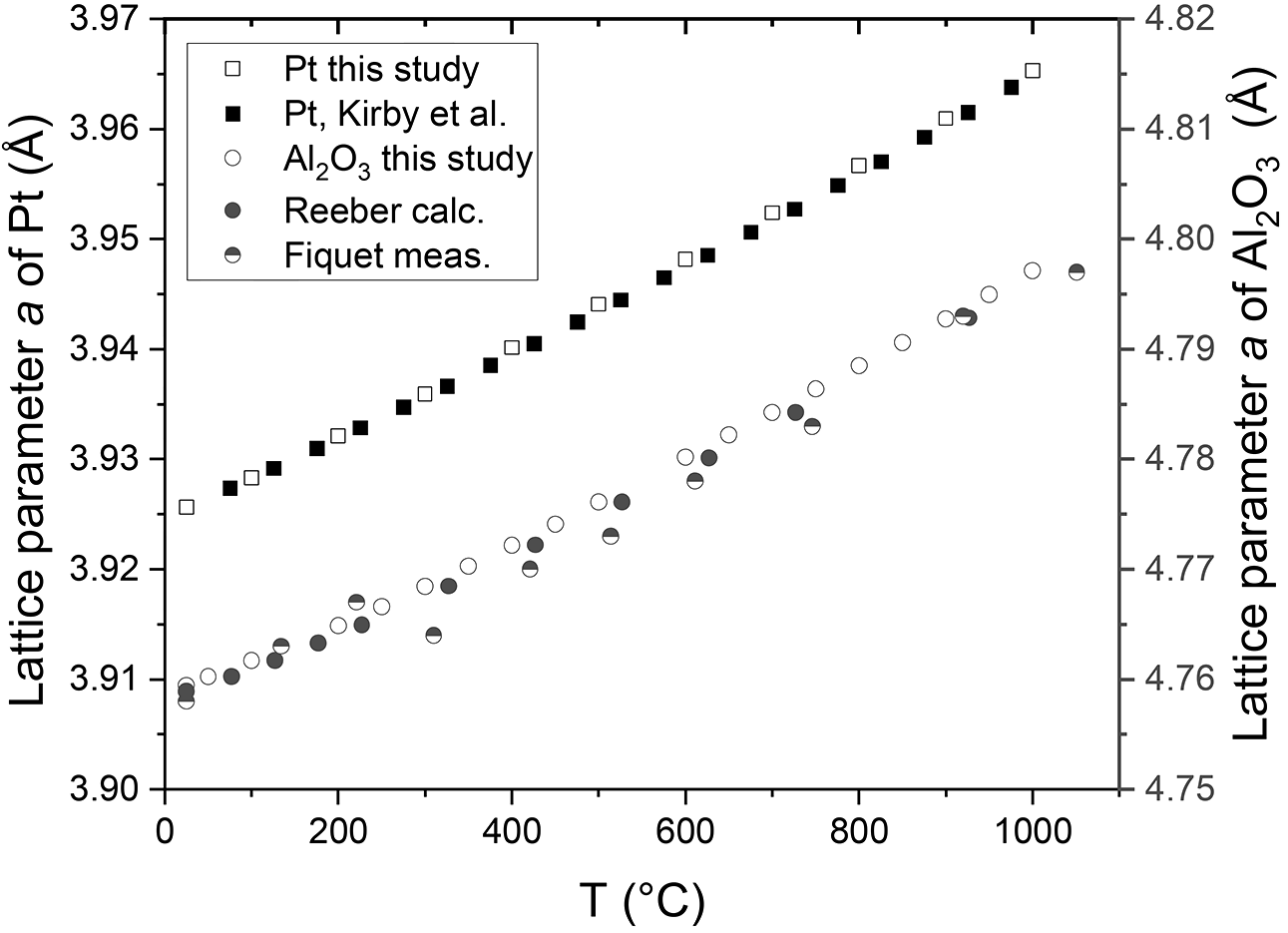
Comparison of measured versus theoretical Pt lattice parameters as a function of temperature. Experimental data were recorded at 50/100°C intervals under an Ar flow rate of 10 Nml min^−1^. Standard deviation values as small as 0.00001 Å are determined for all experimental data.

The temperature at the sample position in the cell was demonstrated by performing *in situ* PXRD experiments on two different standard materials, platinum black (≥99.9%; Sigma-Aldrich) and α-Al_2_O_3_ corundum NIST (∼99.02% ± 1.11%; Sigma-Aldrich). The lattice parameters of Pt and α-Al_2_O_3_ were determined from the Rietveld refinement of PXRD data and compared with the reported values as a function of temperature in the literature. Fig. 4 ▸ shows the lattice parameter of both samples heated from 25°C to 950°C at 50/100°C intervals and under Ar flow of 10 Nml min^−1^ as a function of the recorded temperature of the TC. For comparison, the lattice parameters of platinum are also calculated from its thermal expansion coefficient as reported by Kirby (1991 ▸), while the parameter *a* of α-Al_2_O_3_ is calculated according to first-principle calculations (Reeber & Wang, 2000 ▸) and previous PXRD results (Fiquet *et al.*, 1999 ▸). As shown in Fig. 4 ▸, both platinum and Al_2_O_3_ lattice parameters are in excellent agreement with the theoretical lattice parameter values (Kirby, 1991 ▸; Reeber & Wang, 2000 ▸; Fiquet *et al.*, 1999 ▸). The difference between the measured temperature by the TC and the determined sample temperature derived from the platinum lattice parameter, used as internal temperature calibration for the system, is below 20°C in the full temperature range. Thus, the cell and T-control system provides a very accurate temperature in the sample zone.

### 3.2. De­hydroxy­lation and carbonation of Mg(OH)_2_ in pure CO_2_

To demonstrate the performance of the setup under elevated pressure and temperature, the following de­hydroxy­lation [equation (1)] and carbonation [equation (2)] reactions of Mg(OH)_2_ were investigated:

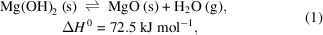




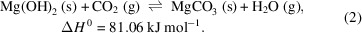

These two reactions have been reported to be temperature- and pressure-dependent (Pfeiffer, 2011 ▸; Fagerlund *et al.*, 2012 ▸).

**Figure 5:**
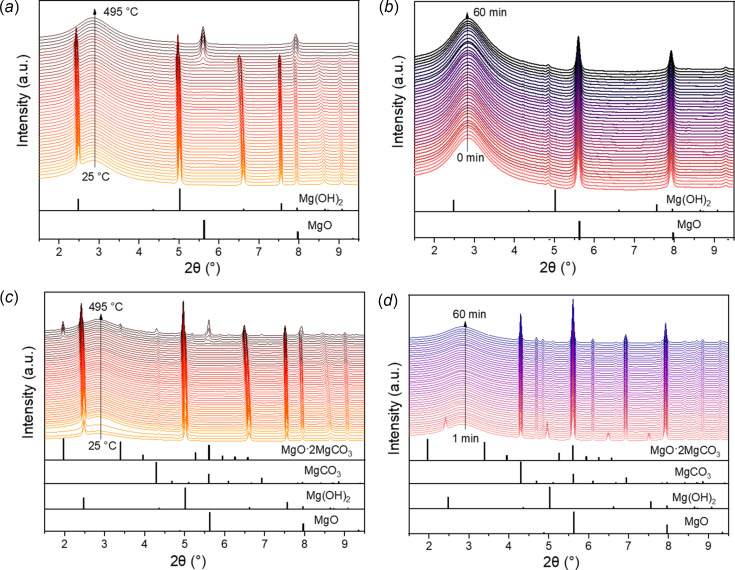
*In situ* PXRD patterns (the wavelength of the X-rays is 0.207286 Å) of pressurized Mg(OH)_2_ in pure CO_2_ using a flow rate of 25 mL min^−1^ during heating after stopping the gas flow at 495°C (*a*, *c*) and holding for 60 min (*b*, *d*) at 1.5 bar (*a*, *b*) and at 35 bar (*c*, *d*). The calculated reference patterns of Mg(OH)_2_ (PDF # 00–044–1482), MgO (PDF # 00–045–0946), MgCO_3_ (PDF # 01–086–2348) and MgO·2MgCO_3_ (PDF # 00–031–0804) are shown at the bottom.

Mg(OH)_2_ powder was filled into a quartz capillary, and the system was first purged with pure CO_2_ for 10 min to remove any residual air; then, the pressure was increased to the target pressure (*i.e.* 1.5 bar and 35 bar) using a total gas flow of 25 mL min^−1^. After pressure stabilization in the system, the temperature was increased to 495°C at 10°C min^−1^ without gas flow to simulate equilibrium conditions. Temperature and pressure were then kept constant for 1 h while changes in the crystal structure were monitored by acquiring one pattern every 30 s; the results are displayed in Fig. 5 ▸. As shown in Figs. 5 ▸(*a*) and 5(*b*), although Mg(OH)_2_ starts transforming first into MgO during heating in both experiments, the de­hydroxy­lation process starts at ∼410°C at 1.5 bar and shifts to a higher temperature (∼450°C) at 35 bar. These results are in line with previous studies that reported the increase in the de­hydroxy­lation temperature of Mg(OH)_2_ by 40–45°C by increasing the pressure from 1 to 35 bar using N_2_ as pressurizing gas. However, the de­hydroxy­lation starts at a slightly higher temperature in our experiments, which agrees with previous studies reported that Ca(OH)_2_ and Mg(OH)_2_ become stable at significantly higher temperatures in the presence of CO_2_ (Fagerlund *et al.*, 2012 ▸; Materić *et al.*, 2010 ▸).

Shortly after reaching 495°C at 1.5 bar, the Mg(OH)_2_ has fully reacted to MgO, and no other reaction product is formed. In contrast, the formed MgO during heating at 35 bar is immediately reacted with CO_2_ to form oxymagnesite MgO·2MgCO_3_, which is usually observed at relatively high pressures during the carbonation of Mg(OH)_2_ (Fagerlund *et al.*, 2012 ▸). This phase disappears after holding the temperature at 495°C for 2 min while the full transformation of the remaining Mg(OH)_2_ phase into MgO and MgCO_3_ takes place after 15 min. Rietveld refinement analysis reveals that the recovered sample at room temperature after carbonation at 495°C and 35 bar for 60 min is composed of 43.6 ± 0.9 wt% MgO and 56.4 ± 1.1 wt% MgCO_3_. These weight fraction values agree with previously reported values at the same temperature and pressure conditions (Fagerlund *et al.*, 2012 ▸), confirming that the pressure in the cell is equal to the set pressure by the pressure control system.

### 3.3. Combined *in situ* PXRD and XAFS study

**Figure 6:**
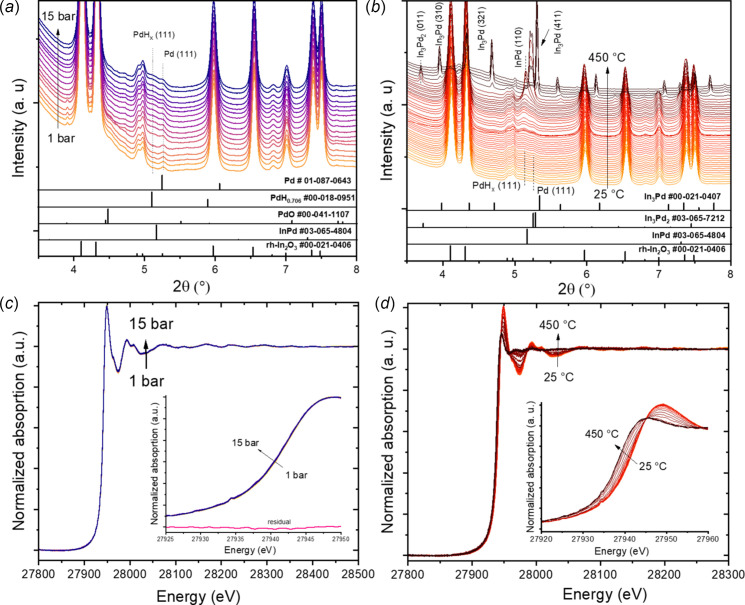
(*a*, *b*) *In situ* PXRD patterns (the wavelength of the X-rays is 0.207286 Å) and (*c*, *d*) In *K*-edge XANES of PdO/rh-In_2_O_3_ material obtained during pressurizing using pure H_2_ up to 15 bar (*a* and *c*), followed by heating at 15 bar from rt to 450°C (*b* and *d*). The residual in the inset in (*c*) highlights that no significant changes in the edge position can be observed during pressurizing.

We studied the structural and chemical changes of reactive metal-support interaction (RMSI) effects occurring on PdO/rh-In_2_O_3_ material during H_2_ reduction separately with PXRD and XAFS using the same cell. The data generated with both techniques can be combined to obtain a more thorough description of a catalyst *in situ*. This RMSI results in the formation of irreversible intermetallic compounds (IMCs) in the PdO/rh-In_2_O_3_ system, influencing its catalytic and gas-sensing properties (Ovsianytskyi *et al.*, 2023 ▸). Our previous *in situ* synchrotron PXRD studies showed that 5 wt% PdO-loaded rh-In_2_O_3_ undergoes a sequence of phase transformations upon heating under a pure H_2_ atmosphere at 1 bar and up to 500°C (Ovsianytskyi *et al.*, 2023 ▸; Schlicker *et al.*, 2018*b* ▸). In this experiment we studied the influence of H_2_ on the sequence of these phase transformations. Fig. 6 ▸ shows the *in situ* PXRD patterns and In *K*-edge X-ray absorption near-edge structure (XANES) for PdO/rh-In_2_O_3_ material obtained during pressurizing using pure H_2_ with flow rate of 25 mL min^−1^ up to 15 bar, followed by heating from room temperature to 450°C at 15 bar after decreasing the flow rate of H_2_ to 5 mL min^−1^. *In situ* PXRD results [Fig. 6 ▸(*a*)] reveal the formation of the PdH_*x*_ phase with increasing pressure of H_2_ to 15 bar at rt, which can be explained by the hydrogenation of Pd metal present in the sample. However, no structural and chemical changes are observed for the rh-In_2_O_3_ phase at these conditions, as indicated from *in situ* PXRD and XAFS results [Fig. 6 ▸(*b*)]. At 15 bar, the interaction between Pd and rh-In_2_O_3_ starts with increasing the temperature, leading to the formation of IMCs such as InPd (at 210°C), In_3_Pd_2_ (260°C) and In_3_Pd (340°C). At 15 bar and 400°C, only the In_3_Pd phase and liquid In metal (recrystallized during cooling) are present in the sample. It is worth noting that our previous *in situ* PXRD study on the same material showed that, at 1 bar of pure H_2_, a sequence of PdO reduction to Pd metal followed by PdH_0.706_ hydride formation and subsequently InPd and In_3_Pd_2_ IMC formation occurred between 30°C and 500°C (Ovsianytskyi *et al.*, 2023 ▸; Schlicker *et al.*, 2018*b* ▸). According to the In–Pd phase diagram, InPd and In_3_Pd_2_ are among the thermodynamically most stable compounds, thus it can be expected that these two phases are predominantly formed at 1 bar. The In_3_Pd phase was not observed at 1 bar in our previous work in the temperature range 30–500°C, suggesting the metastability of this IMC. Thus, increasing the pressure to 15 bar could stabilize the formation of this phase.

The *in situ* PXRD results are in perfect agreement with the XAFS results [Fig. 6 ▸(*d*)], which reveal the shift of the In *K*-edge position to lower energies upon heating due to the reduction of rh-In_2_O_3_ and the formation of IMCs. Moreover, the normalized In *K*-edge XANES spectra at 15 bar and 400°C agree with that of metallic indium (Maeno *et al.*, 2019 ▸). These results confirm the applicability of our setup for both *in situ* synchrotron PXRD and XAFS experiments, allowing for investigating the structural and chemical states of several material systems in a single environment for different applications.

## 4. Conclusion

A setup and cell for the *in situ/operando* characterization with PXRD and XAFS under high temperature, elevated pressure and controllable gas atmosphere has been designed, constructed and demonstrated at two beamlines of the DESY/German synchrotron in Hamburg. The cell acts as plug-flow fixed-bed reactor suitable for monitoring chemical reactions on heterogeneous catalysts and uses two infrared lamps and an SiC tube as an ultra-fast and robust heating system. We demonstrated the functionality of this device in three steps: the heating capability and temperature accuracy based on Pt and Al_2_O_3_ standards; a MgO carbonatization experiment performed up to 35 bar showing the capacity of operating at elevated pressure; a Pd/rh-In_2_O_3_ system studied both with *in situ* PXRD and XAFS. The cell and setup hereby shown are suitable for the characterization of heterogeneous catalysts (with special focus on CO_2_ hydrogenation).

## Supplementary Material

Supporting Figures S1, S2 and S3. DOI: 10.1107/S1600577523009591/vl5016sup1.pdf

## Figures and Tables

**Table 1:** System operating window

Parameter	Minimum	Maximum	Comment
Temperature (°C)	rt	1000	–
Pressure (bar, abs)	1	50	–
Total gas flow (NmL min^−1^)	1.5	73.5	For a precise controllable range of 2% to 98% of the total flow rate
H_2_ inlet concentration (%)	0	100	–
Heating rate (°C s^−1^)	0.167	20	Applied to the rt–500°C interval [see Fig. 3 ▸(*a*)]
Cooling rate (°C s^−1^)	−0.167	−20	No active cooling: cooling rate strongly depends on the temperature gradient cell versus environment [see Fig. 3 ▸(*a*)]
Pressurizing rate (bar min^−1^)	0.04	3	Depressurizing can be performed at a higher rate
