# Supplementary material for: In situ/operando plug-flow fixed-bed cell for synchrotron PXRD and XAFS investigations at high temperature, pressure, controlled gas atmosphere and ultra-fast heating
Source: J Synchrotron Radiat. 2024 Jan 1;31(Pt 1):77–84. doi: 10.1107/S1600577523009591 (PMC10833430; doi:10.1107/S1600577523009591)
Supplement: Supplementary file 1 [file s-31-00077-sup1.pdf]

JOURNAL OF SYNCHROTRON RADIATION

Volume 31 (2024)

Supporting information for article:

In situ/operando plug-flow fixed-bed cell for synchrotron PXRD and XAFS investigations at high temperature, pressure, controlled gas atmosphere and ultra-fast heating

Benjamin Bischoff, Maged F. Bekheet, Emiliano Dal Molin, Sebastian Praetz, Birgit Kanngießer, Reinhard Schomäcker, Martin Etter, Henrik S. Jeppesen, Akhil Tayal, Aleksander Gurlo and Albert Gili

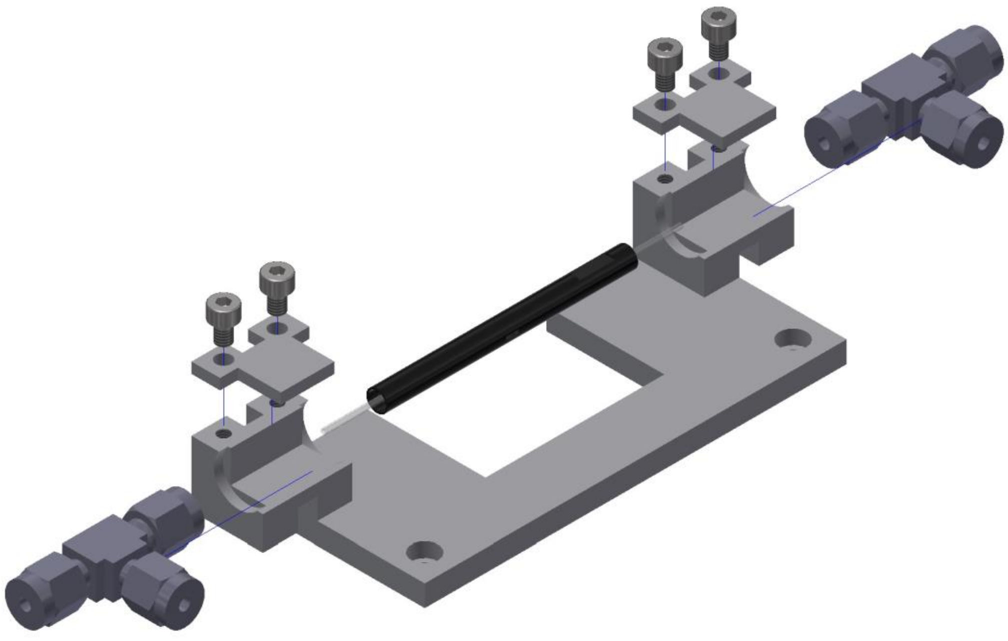

Figure S1 Explosion view of the tube assembly with T-pieces and SiC tube.

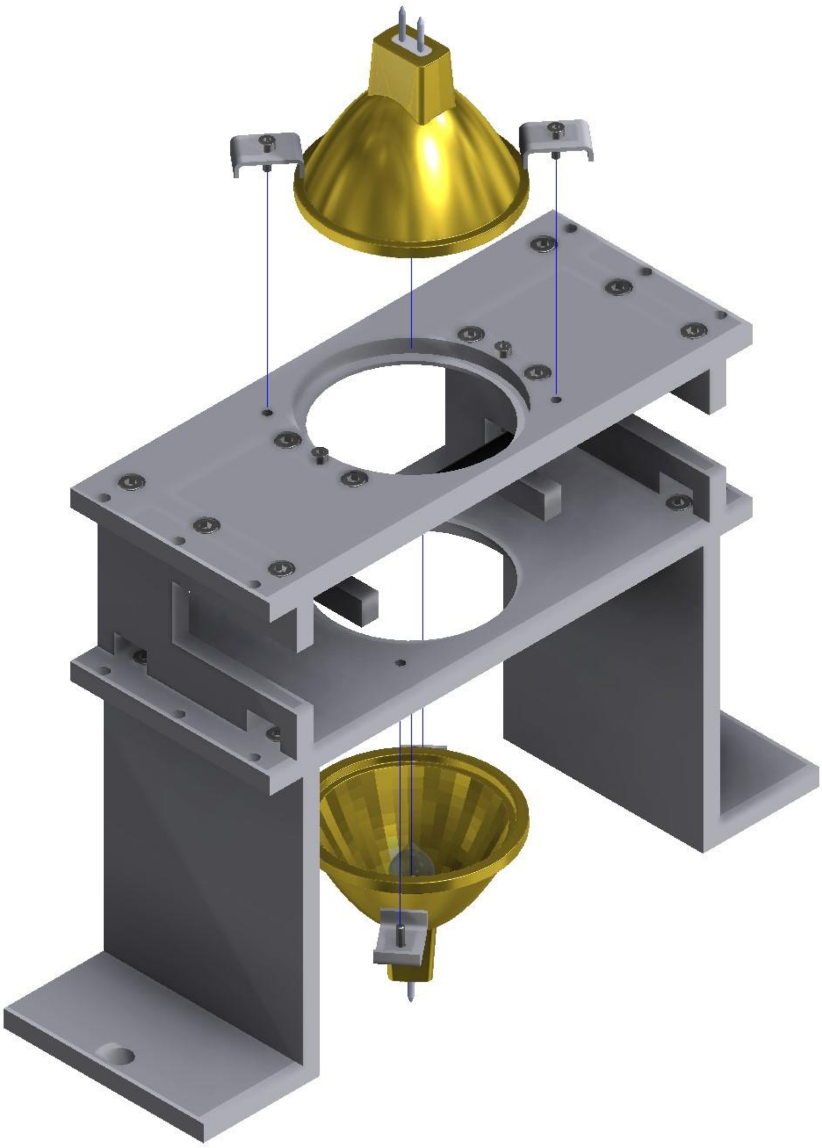

Figure S2 Explosion view of the cell assembly with IR lamps.

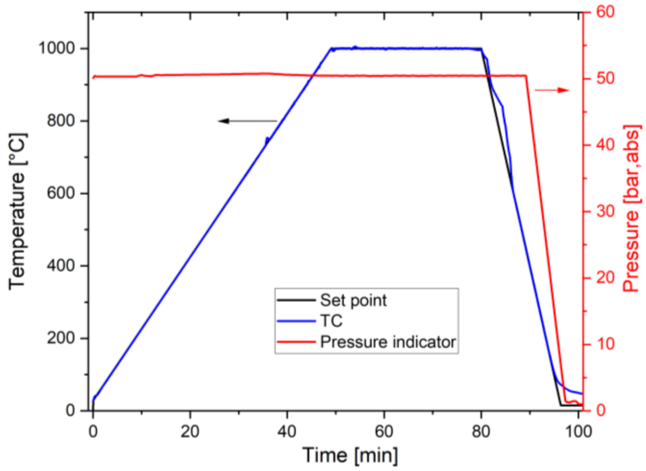

Figure S3 Cell temperature response (TC) tracking the set point (rt-1000 °C, hold at 1000 °C for 30 min and 1000 °C-rt) at 50 bar (abs) as recorded by the PI P-01. Heating rate was 20 °C·min^{-1}; cooling rate was 60 °C·min^{-1} ; the cell was depressurized at 6 bar·min^{-1}. The cell had been pressurized under Argon.
